# Accelerated Biological Aging, Genetic Susceptibility, and the Risk of Abdominal Aortic Aneurysm: A Prospective Cohort Study

**DOI:** 10.31083/RCM46778

**Published:** 2025-12-04

**Authors:** Yiyang Tang, Xinyi Zhou, Qin Chen, Zaixin Yu, Mukamengjiang Juaiti, Lihuang Zha

**Affiliations:** ^1^Department of Cardiology, Beijing Anzhen Hospital, Capital Medical University, 100029 Beijing, China; ^2^National Clinical Research Center for Cardiovascular Diseases, Beijing Anzhen Hospital, Capital Medical University, 100029 Beijing, China; ^3^Department of Cardiology, Xiangya Hospital, Central South University, 410008 Changsha, Hunan, China; ^4^Department 5 of Cardiology, Affiliated Hospital of Traditional Chinese Medicine of Xinjiang Medical University, 830000 Urumqi, Xinjiang Uygur Autonomous Region, China; ^5^National Clinical Research Center for Geriatric Disorders, Xiangya Hospital, Central South University, 410008 Changsha, Hunan, China

**Keywords:** biological aging, genetic susceptibility, abdominal aortic aneurysm, UK Biobank

## Abstract

**Background:**

Biological age (BA) more accurately reflects the true ageing status of the body compared with chronological age. While biological aging is associated with various cardiovascular diseases, the relationship between BA and abdominal aortic aneurysms (AAAs) remains unclear.

**Methods:**

This study utilized data from the UK Biobank for analysis. Telomere length (TL) and BA acceleration, calculated using the Klemera-Doubal method (KDM) and phenotypic age (PhenoAge) methods, were used as surrogate measures of biological aging. Cox regression was primarily performed to explore the association between biological aging and AAA risk. Genetic susceptibility was assessed by constructing a polygenic risk score (PRS).

**Results:**

This study included 311,646 participants with a median age of 58 years. A total of 1339 new cases of AAA (4.33‰) were reported over a median follow-up period of 12.54 years. Each standard deviation (SD) increase in TL was associated with a 17% decreased risk of AAA (hazard ratio (HR) = 0.83, 95% confidence interval (CI) = 0.79–0.88). Each SD increase in BA acceleration in the KDM was associated with a 21% increased risk (HR = 1.21, 95% CI = 1.12–1.29), and and each SD increase in acceleration in the PhenoAge method was associated with a 40% increased risk (HR = 1.40, 95% CI = 1.32–1.48). These associations were independent of genetic risk, as assessed by the PRS, and a joint effect on AAA occurrence was observed. Additionally, we identified a sex-specific modification in the association between telomere shortening and AAA risk, with a significant association observed exclusively in men.

**Conclusions:**

Accelerated biological aging was longitudinally associated with an increased risk of AAA, suggesting that BA may be a significant factor and a potential biomarker for AAA.

## 1. Introduction 

Abdominal aortic aneurysm (AAA) is the most common form of aortic aneurysm, characterized by the permanent and localized dilation of the abdominal aorta [1, 2]. It is often asymptomatic until rupture, a catastrophic event with a mortality rate of up to 80%, highlighting its significant public health burden [3]. Therefore, identifying and validating biomarkers associated with the occurrence of AAA is of significant public health importance, as it can enhance early diagnosis, improve management strategies, and ultimately optimize patient outcomes [4].

AAA is a degenerative disease associated with ageing [5]. Traditionally, chronological age has been used to assess an individual’s level of ageing and disease risk [6]. However, chronological age has limitations, as individuals of the same or similar chronological age can exhibit significant differences in functional status and ageing processes due to factors such as genetics, environment, and lifestyle [7]. Recently, biological age has been proposed as a more comprehensive and accurate measure of ageing, incorporating indicators such as telomere length (TL), the Klemera-Doubal method biological age (KDM-BA), and phenotypic age (PhenoAge) [8]. Previous studies have shown that increased biological age is significantly associated with higher mortality and elevated risks of various cardiovascular diseases, including heart failure [9, 10]. However, the relationship between biological ageing and the incidence of AAA remains unclear. AAA has a genetic predisposition, and genome-wide association studies (GWAS) have identified several single-nucleotide polymorphisms (SNPs) linked to the risk of AAA [11, 12]. The interaction between genetic susceptibility and biological ageing in relation to the risk of AAA has not yet been fully explored.

This study uses a prospective cohort from the UK Biobank to examine the longitudinal associations between biological ageing and the risk of AAA, and to investigate the joint effects and interactions between genetic susceptibility and biological ageing on the long-term risk of AAA.

## 2. Materials and Methods

### 2.1 Study Population

The data used in this study were obtained from the UK Biobank (ID: 107175). The UK Biobank is a large-scale prospective cohort study that recruited over 500,000 volunteers across the United Kingdom between 2006 and 2010 [13]. This study received ethical approval from the North West Multi-Centre Research Ethics Committee (Ref: 11/NW/0382), and all participants provided informed consent by signing a consent form. This study was reported in accordance with the STROBE guidelines.

Of the 502,163 participants in the UK Biobank, we excluded 460 participants with baseline AAA, 177,504 participants missing biomarkers required for the KDM-BA and PhenoAge algorithms, 11,531 participants missing TL measurements, and 1022 participants with missing covariate data. A total of 311,646 participants were included in the final analysis. The participant selection process is shown in **Supplementary Fig. 1**.

### 2.2 Study Outcomes

The outcome variable of this study was the occurrence of AAA, which was determined based on hospital admission and death registration records (**Supplementary Table 1**). The diagnosis of AAA was identified using the Tenth Revision of the International Classification of Diseases (ICD-10 codes: I71.3, I71.4) and the Office of Population, Censuses and Surveys: Classification of Interventions and Procedures (OPCS-4 codes: L18*, L19*, L254, L27*, L28*, L464), as used in prior studies [14]. The study period began when participants entered the cohort and ended upon the diagnosis of AAA, loss to follow-up, death, or completion of follow-up, whichever occurred first. The follow-up cut-off date was September 30, 2021, for England and Wales, and October 31, 2021, for Scotland.

### 2.3 Measurement of TL

TL was measured using a multiplex quantitative polymerase chain reaction method, with samples obtained from peripheral blood leukocytes. Specifically, TL was quantified relative to the ratio of telomere repeat copy number to single-copy gene (T/S), and the data were log-transformed and Z-standardized. Detailed information on the measurement and adjustment of TL can be found in previous studies [15].

### 2.4 Calculation of KDM-BA and PhenoAge

The KDM-BA and PhenoAge algorithms [16, 17] are widely recognized biological age measures based on clinical parameters. These algorithms were developed using optimal training models derived from the National Health and Nutrition Examination Survey (NHANES) and have been further validated with data from the UK Biobank. Using the R package “BioAge” (https://github.com/dayoonkwon/BioAge), KDM-BA was calculated based on forced expiratory volume in one second, systolic blood pressure, and seven blood chemistry parameters: albumin, alkaline phosphatase, blood urea nitrogen, creatinine, C-reactive protein (CRP), glycated hemoglobin, and total cholesterol. PhenoAge was calculated using nine hematological indicators: albumin, alkaline phosphatase, creatinine, CRP, glucose, mean corpuscular volume, red cell distribution width, white blood cell count, and lymphocyte percentage. We then regressed the calculated biological age values against the chronological age at the time of biomarker measurement and computed the residuals [18]. These residuals were termed “age acceleration” (AA) values, which were used to assess biological ageing. The AA values were then standardized with a mean of 0 and a standard deviation of 1 for comparison in subsequent analyses. Further details can be found in **Supplementary Method 1**.

### 2.5 Covariates

The covariates considered in this study included age, sex, ethnicity, education level, Townsend Deprivation Index (TDI), CRP, body mass index (BMI), smoking and alcohol consumption status, physical activity, sleep and dietary patterns, as well as a medical history of hyperlipidemia, hypertension, and diabetes. Detailed definitions and descriptions of these covariates can be found in **Supplementary Method 2** and **Supplementary Tables 2,3**.

### 2.6 Statistical Analysis

Based on the Kolmogorov-Smirnov test (all *p*-values < 0.001) and quantile-quantile (Q-Q) plots, the continuous variables—including age, TDI, BMI, and CRP—were found not to follow a normal distribution and were therefore expressed as medians [interquartile range (IQR)]. Group comparisons were performed using the Mann-Whitney U test or Kruskal-Wallis H test. Categorical variables were represented as frequencies (percentages), and group comparisons were conducted using chi-square tests.

TL, KDM-BA, and PhenoAge acceleration were treated as continuous variables or categorized based on quartiles, and their associations with AAA occurrence were assessed using Cox proportional hazards models, with results presented as hazard ratios (HR) and 95% confidence intervals (95% CI). The Schoenfeld residual test confirmed no violations of the proportional hazards assumption. Model 1 adjusted for age, sex, ethnicity, education level, and TDI. Model 2 further adjusted for smoking and drinking status, physical activity, sleep and dietary patterns, BMI, CRP, and medical history of hypertension, hyperlipidemia, and diabetes. Additionally, based on Cox regression Model 2, a restricted cubic spline (RCS) with three knots was used to analyze the dose-response relationship between biological ageing and AAA risk.

We included only participants of White ancestry and constructed a polygenic risk score (PRS) to assess individuals’ genetic susceptibility to AAA [19] (**Supplementary Method 3**). We then evaluated the joint effect of genetic susceptibility and biological ageing on the risk of AAA. Participants were categorized into 12 groups based on their levels of biological ageing and genetic susceptibility. Using the group with the lowest levels of both biological ageing and genetic susceptibility as a reference, we estimated the HR and 95% CI for AAA occurrence in the other groups, after adjusting for covariates in Model 2. Stratified analyses were conducted to assess the separate effect of biological ageing on the incidence of AAA at different levels of genetic susceptibility. The likelihood ratio test was used to evaluate the interaction between biological ageing and genetic susceptibility.

Subgroup analyses were also conducted to assess potential modifying effects on the association between biological ageing and the risk of AAA. Sensitivity analyses included: (1) Excluding participants who developed AAA within the first year of follow-up; (2) Using the Fine-Gray model to account for mortality as a competing risk. All analyses were performed in R (version 4.3.2, R Foundation for Statistical Computing, Vienna, Austria), with *p*-values < 0.05 considered statistically significant.

## 3. Results

### 3.1 Baseline of the Participants

This study included a total of 311,646 participants (**Supplementary Fig. 1**), of whom 168,249 (53.99%) were women. The majority of participants were White, comprising 294,574 individuals or 94.52% of the total cohort (**Supplementary Table 4**). The median age was 58 years (IQR = 50–63 years), with median values of KDM-BA and PhenoAge recorded at 49.82 years (IQR = 40.90–58.26) and 45.83 years (IQR = 38.10–52.54), respectively. KDM-BA (r = 0.67) and PhenoAge (r = 0.86) were strongly positively correlated with chronological age, whereas TL showed a weak negative correlation (r = –0.20, **Supplementary Fig. 2**).

Baseline characteristics, stratified by quartiles of TL, PhenoAge acceleration, and KDM-BA acceleration, are summarized in Table 1 and **Supplementary Tables 5,6**. Individuals with longer TL had a higher proportion of women, a higher level of education, higher TDI, lower BMI and CRP, and a higher proportion of those maintaining a healthy lifestyle (never smoking, never drinking, regular physical activity, a healthy sleep pattern, and a healthy diet). They also had a lower proportion of comorbid hypertension, diabetes, and hyperlipidemia. Similarly, participants with higher KDM-BA acceleration and higher PhenoAge acceleration had higher TDI, lower educational attainment, higher CRP levels, and a higher prevalence of comorbidities such as hypertension, diabetes, and hyperlipidemia.

**Table 1.** **Baseline characteristics of study participants grouped by telomere length quartiles**.

Variables		Total	Telomere length				*p* for overall	*p* for trend
			Q1	Q2	Q3	Q4		
N		311,646	77,912	77,911	77,911	77,912		
Age (years)		58.00 [50.00, 63.00]	60.00 [53.00, 65.00]	58.00 [51.00, 63.00]	57.00 [49.00, 62.00]	55.00 [47.00, 61.00]	<0.001	<0.001
Women, n (%)		168,249 (53.99)	37,583 (48.24)	40,641 (52.16)	43,183 (55.43)	46,842 (60.12)	<0.001	<0.001
White, n (%)		294,574 (94.52)	74,643 (95.80)	74,127 (95.14)	73,538 (94.39)	72,266 (92.75)	<0.001	<0.001
TDI		–2.20 [–3.67, 0.39]	–2.24 [–3.68, 0.34]	–2.22 [–3.68, 0.33]	–2.19 [–3.67, 0.39]	–2.13 [–3.63, 0.51]	<0.001	<0.001
Education, n (%)							<0.001	<0.001
	College	101,091 (32.44)	22,490 (28.87)	24,385 (31.30)	25,913 (33.26)	28,303 (36.33)		
	High school	35,071 (11.25)	7940 (10.19)	8696 (11.16)	8918 (11.45)	9517 (12.22)		
	Middle school	67,420 (21.63)	16,899 (21.69)	16,990 (21.81)	16,993 (21.81)	16,538 (21.23)		
	Others	108,064 (34.68)	30,583 (39.25)	27,840 (35.73)	26,087 (33.48)	23,554 (30.23)		
BMI, kg/m^2^		26.68 [24.11, 29.79]	26.98 [24.39, 30.07]	26.78 [24.20, 29.86]	26.64 [24.06, 29.76]	26.33 [23.81, 29.44]	<0.001	<0.001
CRP, mg/L		1.30 [0.64, 2.67]	1.41 [0.71, 2.85]	1.32 [0.66, 2.70]	1.27 [0.63, 2.61]	1.19 [0.59, 2.50]	<0.001	<0.001
Drinking status, n (%)							<0.001	<0.001
	Never	13,303 (4.27)	3145 (4.04)	3179 (4.08)	3402 (4.37)	3577 (4.59)		
	Previous	10,545 (3.38)	2786 (3.58)	2578 (3.31)	2595 (3.33)	2586 (3.32)		
	Current	287,103 (92.12)	71,807 (92.16)	71,978 (92.38)	71,750 (92.09)	71,568 (91.86)		
Smoking status, n (%)							<0.001	<0.001
	Never	171,217 (54.94)	40,161 (51.55)	42,177 (54.13)	43,698 (56.09)	45,181 (57.99)		
	Previous	107,241 (34.41)	28,787 (36.95)	27,418 (35.19)	26,162 (33.58)	24,874 (31.93)		
	Current	31,733 (10.18)	8575 (11.01)	7948 (10.20)	7714 (9.90)	7496 (9.62)		
Physical activity, n (%)							<0.001	<0.001
	Regular	122,958 (39.45)	29,800 (38.25)	30,289 (38.88)	31,060 (39.87)	31,809 (40.83)		
	Excessive	75,514 (24.23)	19,175 (24.61)	19,133 (24.56)	18,780 (24.10)	18,426 (23.65)		
	Poor	43,693 (14.02)	10,746 (13.79)	10,956 (14.06)	10,928 (14.03)	11,063 (14.20)		
Sleep pattern, n (%)							<0.001	<0.001
	Healthy	151,320 (48.56)	37,158 (47.69)	37,521 (48.16)	38,006 (48.78)	38,635 (49.59)		
	Intermediate	100,598 (32.28)	25,635 (32.90)	25,319 (32.50)	25,132 (32.26)	24,512 (31.46)		
	Poor	5807 (1.86)	1556 (2.00)	1475 (1.89)	1439 (1.85)	1337 (1.72)		
Diet pattern, n (%)							<0.001	<0.001
	Healthy	117,408 (37.67)	28,452 (36.52)	29,244 (37.54)	29,456 (37.81)	30,256 (38.83)		
	Intermediate	145,882 (46.81)	36,825 (47.26)	36,562 (46.93)	36,512 (46.86)	35,983 (46.18)		
	Poor	35,506 (11.39)	9257 (11.88)	8857 (11.37)	8806 (11.30)	8586 (11.02)		
Medical history, n (%)								
	Hyperlipidemia	144,832 (46.47)	38,831 (49.84)	37,139 (47.67)	35,561 (45.64)	33,301 (42.74)	<0.001	<0.001
	Hypertension	170,827 (54.81)	46,307 (59.44)	43,982 (56.45)	41,630 (53.43)	38,908 (49.94)	<0.001	<0.001
	Diabetes mellitus	17,765 (5.70)	5454 (7.00)	4597 (5.90)	4152 (5.33)	3562 (4.57)	<0.001	<0.001

Abbreviation: TDI, Townsend deprivation index; CRP, C-reactive protein; BMI, body mass index. Note: Q1–Q4 (quartiles) of Z-standardised telomere length: Q1 (<–0.64), Q2 (<0.00, ≥–0.64), Q3 (<0.65, ≥0.00), Q4 (≥0.65).

### 3.2 Association Between Biological Ageing and the Risk of AAA

**Fig. 1.**
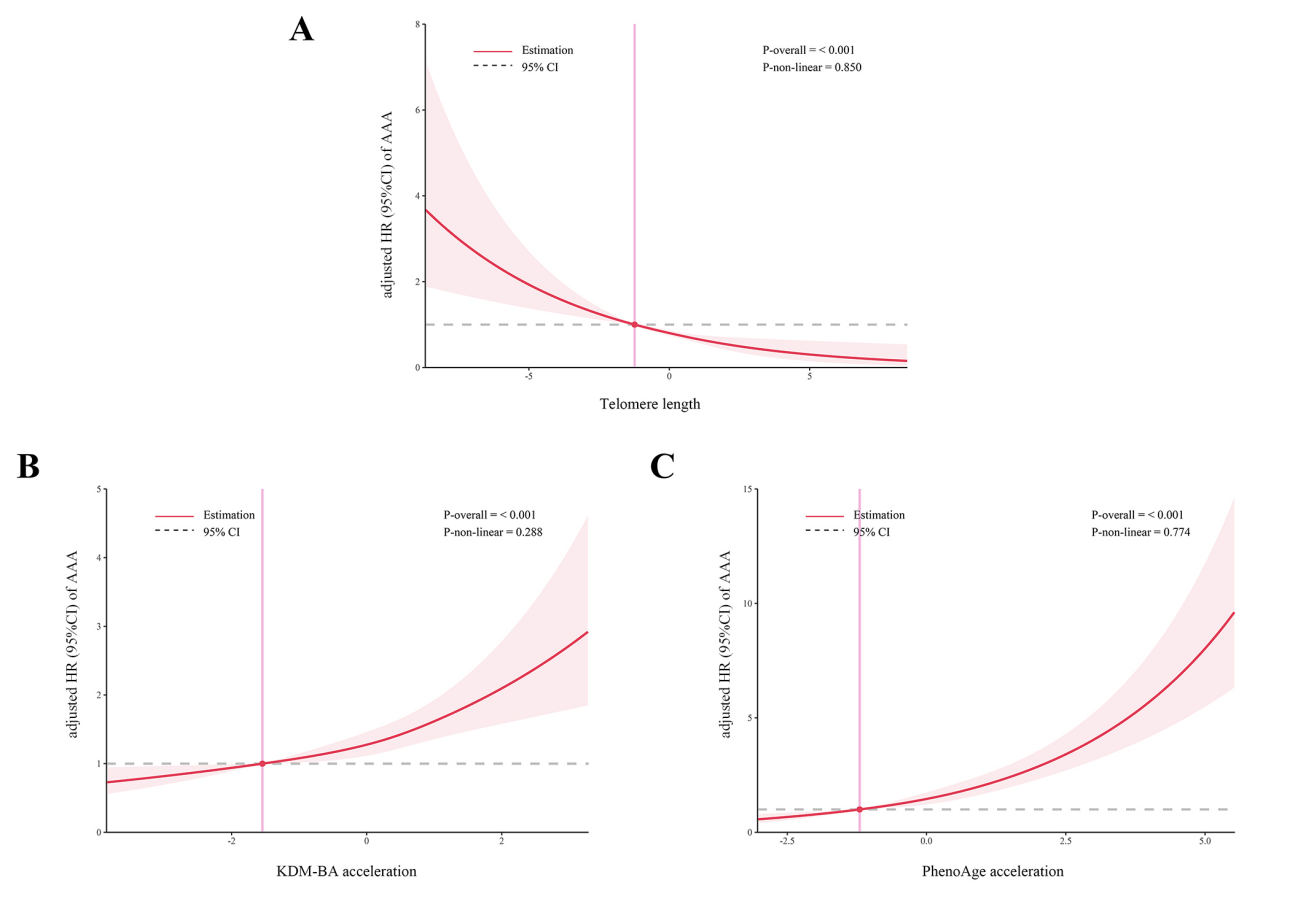
**Association between biological ageing and the risk of AAA using restricted cubic splines model with three knots**. Panels A-C depict the dose-response relationships between telomere length (A), KDM-BA acceleration (B), and PhenoAge acceleration (C) and the risk of AAA, respectively. Abbreviation: AAA, abdominal aortic aneurysm.

During a median follow-up of 12.54 years (IQR = 11.87–13.16), a total of 1339 (4.29‰) incident cases of AAA were reported. Overall, accelerated biological ageing was significantly positively associated with an increased long-term risk of AAA (Table 2). For each standard deviation increase in TL, the risk of AAA decreased by 17% (HR = 0.83, 95% CI = 0.79–0.88); for each standard deviation increase in KDM-BA acceleration, the risk increased by 21% (HR = 1.21, 95% CI = 1.12–1.29); and for each standard deviation increase in PhenoAge acceleration, the risk increased by 40% (HR = 1.40, 95% CI = 1.32–1.48). Participants with longer TL had a significantly lower risk of AAA compared with those with shorter TL, while those with higher KDM-BA acceleration or higher PhenoAge acceleration had a significantly higher risk of AAA compared with those with lower KDM-BA acceleration or lower PhenoAge acceleration. Fig. 1 demonstrates the potential dose-response relationships between biological ageing and the risk of AAA. The results indicated a linear association between TL, KDM-BA acceleration, PhenoAge acceleration, and the occurrence of AAA (*p*-value for non-linearity >0.05).

**Table 2.** **Association between biological ageing and the risk of abdominal aortic aneurysm**.

Variables		Cases/N	Incidence rate*	Model 1		Model 2	
				HR (95% CI)	*p* value	HR (95% CI)	*p* value
Telomere length							
	Q1	545/77,912	5.755	1 (reference)		1 (reference)	
	Q2	360/77,911	3.765	0.83 (0.73, 0.95)	0.006	0.85 (0.75, 0.98)	0.020
	Q3	270/77,911	2.813	0.75 (0.65, 0.87)	<0.001	0.79 (0.68, 0.92)	0.002
	Q4	164/77,912	1.703	0.60 (0.50, 0.72)	<0.001	0.64 (0.54, 0.76)	<0.001
	Continuous	1339/311,646	3.499	0.82 (0.77, 0.86)	<0.001	0.83 (0.79, 0.88)	<0.001
KDM-BA acceleration							
	Q1	420/77,912	4.39	1 (reference)		1 (reference)	
	Q2	455/77,911	4.76	1.61 (1.41, 1.84)	<0.001	1.23 (1.08, 1.42)	0.003
	Q3	165/77,911	1.72	1.93 (1.59, 2.34)	<0.001	1.25 (1.02, 1.53)	0.034
	Q4	299/77,912	3.14	3.18 (2.70, 3.75)	<0.001	1.67 (1.37, 2.02)	<0.001
	Continuous	1339/311,646	3.50	1.53 (1.44, 1.62)	<0.001	1.21 (1.12, 1.29)	<0.001
PhenoAge acceleration							
	Q1	131/77,912	1.35	1 (reference)		1 (reference)	
	Q2	212/77,911	2.20	1.28 (1.03, 1.59)	0.027	1.15 (0.93, 1.44)	0.202
	Q3	335/77,911	3.50	1.81 (1.48, 2.22)	<0.001	1.48 (1.20, 1.82)	<0.001
	Q4	661/77,912	7.09	3.36 (2.78, 4.06)	<0.001	2.15 (1.76, 2.63)	<0.001
	Continuous	1339/311,646	3.50	1.61 (1.54, 1.70)	<0.001	1.40 (1.32, 1.48)	<0.001

*The incidence rate was reported as per 10,000 person-years. Model 1 adjusted for age, sex, ethnicity, Townsend deprivation index, education levels. Model 2 adjusted for model 1 plus body mass index, C-reactive protein, smoking and drinking status, physical activity, sleep and diet patterns, history of hyperlipemia, hypertension, and diabetes mellitus. Abbreviation: KDM-BA, Klemera-Doubal method biological age; PhenoAge, phenotypic age; HR, hazard ratio; CI, confidence interval.

### 3.3 Joint and Interaction Analysis of Biological Ageing and Genetic Predisposition

The results in **Supplementary Table 7** indicated that the constructed PRS score effectively predicted the risk of the occurrence of AAA. After further adjusting for PRS in Models 1 and 2, the significant association between biological ageing indicators and AAA risk persisted (**Supplementary Table 8**).

**Fig. 2.**
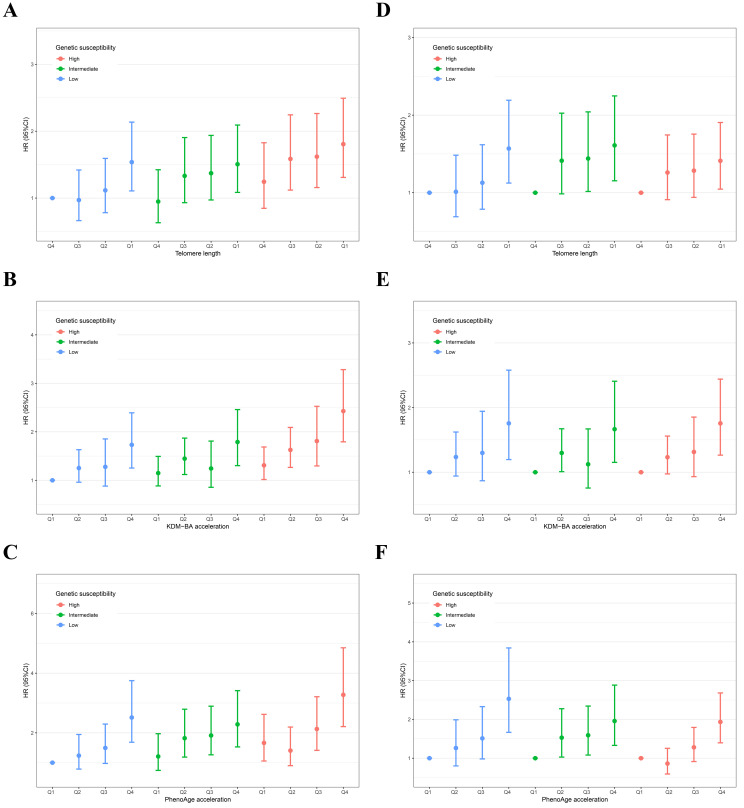
**Separate and joint association of biological ageing with the risk of AAA in individuals with different levels of genetic susceptibility**. Panels A–C show the joint effects of telomere length (A), KDM-BA acceleration (B), and PhenoAge acceleration (C) with genetic susceptibility, respectively; Panels D–F show the separate effects of telomere length (D), KDM-BA acceleration (E), and PhenoAge acceleration (F) across different levels of genetic susceptibility, respectively. The result was presented as HR (95% CI) using the Cox proportional hazards model adjusted for age, sex, ethnicity, Townsend deprivation index, education levels, body mass index, C-reactive protein, smoking and drinking status, physical activity, sleep and diet patterns, history of hyperlipemia, hypertension, and diabetes mellitus.

The joint effects of biological ageing and genetic susceptibility on the risk of AAA are shown in Fig. 2A–C. The risk of AAA increased with accelerated biological ageing and higher levels of genetic susceptibility (*p*-value for trend <0.001). Compared to participants with the lowest PRS and biological ageing, those with the highest PRS and biological ageing exhibited the highest risk of AAA (high PRS and Q1 of TL: HR = 1.81, 95% CI = 1.31–2.49; high PRS and Q4 of KDM-BA acceleration: HR = 2.43, 95% CI = 1.79–3.28; high PRS and Q4 of PhenoAge acceleration: HR = 3.27, 95% CI = 2.21–4.85). At different levels of genetic susceptibility, higher levels of biological ageing were positively associated with the occurrence of AAA (Fig. 2D–F). No significant interaction was observed between genetic susceptibility and biological ageing indicators regarding the risk of AAA (*p*-value for interaction >0.05).

### 3.4 Subgroup and Sensitivity Analyses

As shown in Table 3, subgroup analyses indicated that the effect of longer TL in reducing the incidence of AAA was more significant in men than in women (*p*-value for the interaction between sex and TL = 0.018). For other stratification factors, no significant differences were observed in the associations between biological ageing indicators and AAA occurrence (*p*-value for the interaction >0.05, **Supplementary Tables 9,10**). Sensitivity analyses, including the exclusion of participants who reported AAA in the first year (**Supplementary Table 11**) and the application of Fine-Gray competing risk regression models (**Supplementary Fig. 3**), consistently supported the strong association between high levels of biological ageing and an increased incidence of AAA.

**Table 3.** **Subgroup analysis for the association between telomere length with the risk of abdominal aortic aneurysm**.

Subgroup		Cases/N	Telomere length				*p* for interaction
			Q1	Q2	Q3	Q4	
Age							0.171
	<45	5/32,843	1 (reference)	0.80 (0.09, 7.16)	1.32 (0.22, 7.92)	0.00 (0.00, Inf)	
	≥45, <55	60/89,533	1 (reference)	0.79 (0.43, 1.44)	0.42 (0.19, 0.90)	0.39 (0.18, 0.87)	
	≥45, <55	600/131,278	1 (reference)	0.98 (0.79, 1.20)	0.91 (0.74, 1.14)	0.70 (0.55, 0.90)	
	≥65	674/57,992	1 (reference)	0.77 (0.64, 0.94)	0.65 (0.53, 0.80)	0.61 (0.49, 0.77)	
Age							0.409
	<65	665/253,654	1 (reference)	0.84 (0.70, 1.02)	0.68 (0.55, 0.84)	0.52 (0.41, 0.66)	
	≥65	674/57,992	1 (reference)	0.77 (0.64, 0.94)	0.65 (0.53, 0.80)	0.61 (0.49, 0.77)	
Sex							0.018
	Women	205/168,249	1 (reference)	0.79 (0.55, 1.14)	0.84 (0.58, 1.23)	1.00 (0.68, 1.46)	
	Men	1134/143,397	1 (reference)	0.86 (0.74, 0.99)	0.78 (0.67, 0.92)	0.59 (0.49, 0.72)	
Ethnicity							0.829
	White	1308/294,574	1 (reference)	0.85 (0.74, 0.97)	0.81 (0.70, 0.94)	0.64 (0.54, 0.77)	
	Non-White	31/17,072	1 (reference)	0.68 (0.28, 1.62)	0.61 (0.22, 1.70)	0.50 (0.14, 1.78)	
TDI							0.557
	Low	411/104,004	1 (reference)	0.89 (0.70, 1.13)	0.84 (0.65, 1.10)	0.54 (0.39, 0.76)	
	Moderate	445/103,925	1 (reference)	0.79 (0.62, 1.00)	0.81 (0.63, 1.05)	0.78 (0.58, 1.03)	
	High	483/103,717	1 (reference)	0.89 (0.71, 1.11)	0.72 (0.56, 0.92)	0.60 (0.45, 0.82)	
Education							0.519
	College	223/101,091	1 (reference)	0.78 (0.56, 1.09)	0.89 (0.63, 1.25)	0.55 (0.35, 0.86)	
	High school	109/35,071	1 (reference)	0.84 (0.52, 1.33)	0.87 (0.53, 1.42)	0.42 (0.21, 0.84)	
	Middle school	289/67,420	1 (reference)	1.01 (0.76, 1.35)	0.89 (0.65, 1.22)	0.79 (0.55, 1.14)	
	Others	718/108,064	1 (reference)	0.76 (0.63, 0.92)	0.72 (0.59, 0.88)	0.62 (0.49, 0.78)	
Hypertension							0.647
	No	235/140,819	1 (reference)	0.86 (0.63, 1.18)	0.86 (0.61, 1.21)	0.47 (0.29, 0.77)	
	Yes	1104/170,827	1 (reference)	0.84 (0.73, 0.98)	0.79 (0.67, 0.92)	0.67 (0.56, 0.80)	
Hyperlipidemia							0.266
	No	344/166,814	1 (reference)	0.95 (0.74, 1.23)	0.81 (0.61, 1.09)	0.55 (0.38, 0.81)	
	Yes	995/144,832	1 (reference)	0.84 (0.71, 0.98)	0.75 (0.63, 0.89)	0.69 (0.57, 0.84)	
Diabetes							0.516
	No	1201/293,881	1 (reference)	0.87 (0.75, 1.00)	0.77 (0.66, 0.90)	0.64 (0.54, 0.78)	
	Yes	138/17,765	1 (reference)	0.87 (0.56, 1.34)	0.94 (0.60, 1.47)	0.76 (0.45, 1.26)	

Adjusted for age, sex, ethnicity, TDI, education levels body mass index, C-reactive protein, smoking and drinking status, physical activity, sleep and diet patterns, history of hyperlipemia, hypertension, and diabetes mellitus. Abbreviation: TDI, Townsend deprivation index.

## 4. Discussion

In this study, we utilized the large-scale prospective cohort of the UK Biobank to investigate the association between clinical biomarker-based biological ageing and the long-term risk of AAA. The main findings are as follows: accelerated biological ageing (reflected by shortened TL, higher KDM-BA, and PhenoAge acceleration) was significantly associated with an increased risk of AAA. This association was independent of genetic risk assessed by PRS and demonstrated a joint effect with genetic predisposition. Furthermore, sex appeared to modify the relationship between TL and the risk of AAA, with the association between shortened TL and an increased risk of AAA being significant only in men.

The association between biological ageing and cardiovascular diseases has been increasingly recognized [20, 21], and accurately measuring biological age is key to further studying this relationship [22]. Various indicators for assessing an individual’s biological age have been proposed, each of which is distinctive in reflecting different dimensions and mechanisms of the ageing process. Telomeres are tandemly repeated nucleotide sequences located at the ends of chromosomes, and TL is considered a key indicator of biological ageing [23]. When telomeres shorten to a critical threshold, an irreparable DNA damage response is triggered, leading to the cessation of cell division and, consequently, ageing [24]. Atturu *et al*. [25] showed that, compared to the control group, patients with AAA had shorter leukocyte telomeres, and this telomere shortening was significantly associated with the risk of AAA (odds ratio [OR] = 2.30, 95% CI = 1.28–4.13). However, this was a case-control study with a limited sample size (N = 373), and it did not establish a longitudinal association between TL and the occurrence of AAA. This study partially addressed this issue and provided stronger evidence. We also found an interesting interaction between TL and sex. The association between biological ageing and AAA risk was only significant in men. The remodeling of the extracellular matrix has been found to be more pronounced in the ageing process of men compared to women, as evidenced by increased aortic diameter and stiffness [26]. This may be attributed to the protective effects of estrogen [27]. In addition, several composite biomarker algorithms, such as KDM-BA and PhenoAge, have been proposed in recent years to estimate an individual’s biological age. Compared to single indicators such as TL, these algorithms are more reliable and comprehensive, as they integrate a range of clinical biomarkers and anthropometric data while also considering multiple dimensions, such as metabolism, immunity, inflammation, and organ homeostasis [28]. Furthermore, their high accessibility and affordability further enhance their potential for widespread application.

Biological ageing may contribute to the development of AAA through various mechanisms. Chronic low-grade inflammation is a significant characteristic of ageing and a key mechanism in the development of AAA [29]. Senescent cells secrete a complex array of factors, including pro-inflammatory cytokines, chemokines, growth factors, and matrix metalloproteinases [30]. These factors can recruit various inflammatory cells to infiltrate the vascular wall, creating an inflammatory microenvironment that compromises aortic structural integrity and promotes the occurrence of AAA [31]. Oxidative stress may only partly explain the association between biological ageing and AAA. Research has shown that ageing is related to the dysfunction of nuclear factor E2-related factor 2 in the aorta, exacerbating oxidative stress and increasing sensitivity to reactive oxygen species-mediated damage [32]. The phenotypic transformation of smooth muscle cells is another important mechanism in the development of aortic aneurysms. Under pressure induction, ageing vascular smooth muscle cells secrete fibroblast growth factor 9, causing adjacent normal vascular smooth muscle cells to undergo phenotypic transformation, which may contribute to the formation of AAA [33].

The strength of this study lies in establishing a longitudinal association between biological ageing and the long-term risk of AAA. The large sample size and high-quality data from the UK Biobank prospective cohort ensured the credibility of the study results. In addition, this study is comprehensive, utilizing three different indicators to assess individual biological ageing levels: TL, a key marker of ageing, and KDM-BA and PhenoAge, which are calculated based on multidimensional biomarkers. Furthermore, we also took into account the role of genetic susceptibility in this association. This study also has some limitations. First, as an observational study, it cannot establish causality or clarify the specific mechanisms underlying the association between biological ageing and AAA. Second, biological ageing was assessed solely through baseline data, preventing further exploration of the relationship between longitudinal changes in biological ageing and the risk of AAA. Although we adjusted for multiple confounding factors, the study cannot rule out the potential influence of important confounders not available in the UK Biobank. In addition, since the participants of the UK Biobank were primarily White, the generalizability of the findings to other populations needs further confirmation.

## 5. Conclusions

Biomarker-based biological ageing is significantly associated with the long-term risk of AAA, independent of genetic susceptibility. These findings highlight the potential utility of biological ageing assessments in identifying high-risk populations for AAA, providing valuable insights for its prevention and management.

## Availability of Data and Materials

The data supporting the findings of this study were obtained from the UK Biobank Resource under Application No. 107175. These data can be accessed by submitting an application through the UK Biobank official website (https://www.ukbiobank.ac.uk).

## Supplementary Material

Supplementary material associated with this article can be found, in the online version, at https://doi.org/10.31083/RCM46778.
