# Supplementary material for: Accelerated Biological Aging, Genetic Susceptibility, and the Risk of Abdominal Aortic Aneurysm: A Prospective Cohort Study
Source: Rev Cardiovasc Med. 2025 Dec 4;26(12):46778. doi: 10.31083/RCM46778 (PMC12780995; doi:10.31083/RCM46778)
Supplement: Supplementary file 1 [file 2153-8174-26-12-46778-s1.docx]

**Supplementary Method 1. Calculation of KDM-BA and PhenoAge**

An individual’s KDM-BA prediction represents the chronological age at which their physiology would be approximately normal. The KDM-BA algorithm is derived from a series of regressions of individual biomarkers on chronological age (CA) in a reference population. The equation utilizes data from n number of regression lines of CA regressed on n biomarkers^1^. The formula is:

$${KDM-BA}_{EC}=\frac{\sum_{\text{i}=1}^{n} \left( x_{\text{i}}-q_{\text{i}} \right)\frac{k_{\text{i}}}{s_{\text{i}}^{2}}+ \frac{CA}{s_{BA}^{2}}}{\sum_{\text{i}=1}^{n} \left( \frac{k_{\text{i}}}{s_{\text{i}}} \right)^{2}+ \frac{1}{s_{BA}^{2}}}$$

where x is the value of biomarker i measured for an individual. For each biomarker i, the parameters k, q, and s are estimated from a regression of CA on the biomarker in the reference sample. k, q, and s represent the regression intercept, slope, and root mean squared error, respectively. sBA is a scaling factor equal to the square root of the variance in chronological age explained by the biomarker set in the reference sample. In the BioAge package, the reference sample is NHANES III nonpregnant participants aged 30–75 years. Algorithm parameters are estimated separately for men and women.

The PhenoAge algorithm is based on multivariate analysis of mortality hazards. The original PhenoAge algorithm was developed through elastic-net Gompertz regression of mortality on 42 biomarkers from the NHANES III dataset^2^. This analysis selected nine biomarkers, as described above, along with CA. The formula is as follows:

$$\text{PhenoAge}=141\cdot50225+\frac{\ln\left[ -0\cdot00553\times ln(1-mortality risk) \right]}{0\cdot090165}$$

where

$$\text{mortality risk}=1-e^{-e^{{xb}_{\frac{\left[ \text{exp}({120}_{x\gamma})-1 \right]}{\gamma}}}}$$

$$\gamma=\text{0·0076927}$$

$$xb\text{=}-\text{19·907}-\text{0·0336}+\text{albumin}+\text{0·0095}\times\text{creatinine}+\text{0·1953}\times\text{glucose}+\text{0·0954}\times\ln\left( C\text{-reactive protein} \right)-\text{0·012}\times\text{lymphocyte percentage}+\text{0·0268}\times\text{mean corpuscular volume}+\text{0·3306}\times\text{red cell distribution width}+\text{0·00188}\times\text{alkaline phosphatase}+\text{0·0554}\times\text{white blood cell count}+\text{0·0804}\times\text{chronological age}$$

**Supplementary Method 2. Definition of covariates**

At baseline, information on age, sex, ethnicity, education level, Townsend Deprivation Index (TDI), smoking status, alcohol consumption, dietary habits, sleep quality, and physical activity was collected using touchscreen questionnaires. Ethnicity was categorized as White or Other, and education level was classified into college, high school, middle school, or other categories. TDI, an indicator of material deprivation, was utilized to comprehensively evaluate living conditions, with higher scores reflecting greater levels of deprivation. Smoking and alcohol consumption were categorized into three groups: never, previous, and current. Metabolic Equivalent Task (MET) was calculated according to the guidelines of the International Physical Activity Questionnaire (IPAQ), with participants grouped into three categories: poor physical activity (<600 MET-min/week), regular physical activity (600-3000 MET-min/week), and excessive physical activity (>3000 MET-min/week) ^3^. Sleep patterns were assessed using a healthy sleep score, which evaluated various aspects of sleep quality, including sleep duration, sleep chronotype, insomnia, snoring, and daytime sleepiness^4^. Dietary habits were evaluated using a healthy diet score, which considered the intake or frequency of consumption of various foods, including vegetables, fruits, fish, red meat, and processed meat^5^. Both healthy diet and sleep scores ranged from 0 to 5 points, with scores of 0-1, 2-3, and 4-5 defined as poor, intermediate, and healthy patterns, respectively. Details regarding the construction of the healthy diet and sleep scores were provided in **Supplementary Table 2**. Body mass index (BMI) was calculated using height and weight measured during the initial assessment center visit, with the formula: weight (kg) divided by height squared (m²). C-reactive protein (CRP) levels were measured using high-sensitivity immunoturbidimetric analysis on a Beckman Coulter AU5800 platform. Disease diagnoses were obtained from "First Occurrences" data and verified using the corresponding ICD-10 codes (**Supplementary Table 1**). Hypertension was defined as the use of antihypertensive medication, a physician’s diagnosis of hypertension, or a mean blood pressure measurement (average of two readings) of ≥140/90 mmHg. Diabetes was defined as a physician’s diagnosis of diabetes, use of antidiabetic medication, or a glycated hemoglobin (HbA1c) level >6.5%. Hyperlipidemia was defined as a physician’s diagnosis of hyperlipidemia, use of lipid-lowering medication, or a low-density lipoprotein cholesterol (LDL-C) level >4.0 mmol/L^6^.

**Supplementary Method 3. Construction of polygenic risk score**

The base data for constructing the polygenic risk score (PRS) was sourced from the FinnGen Genome-Wide Association Study (GWAS), while the target data was extracted from the UK Biobank genetic dataset^7^. The FinnGen study on AAA included 4,083 cases and 41,697 controls. All participants from both FinnGen and the UK Biobank were White, with no overlap between the two cohorts. Data cleaning was performed using Plink v1.9 and the "tidyverse" package in R. The PRS was generated using PRSice-2.1 software. The PRS was computed using *p*-value thresholds ranging from 1 to 5×10^-8 based on the base data, and linkage disequilibrium clumping was applied to the target data using the default settings (clumping distance of 250 Kb and an R^2 threshold of 0.1). The default model of the PRS algorithm was chosen, and the results of all variants were summed to generate the final PRS score. The PRS score was then standardized through z-score transformation. The summary results of the independent single nucleotide polymorphisms used for PRS construction were presented in **Supplementary Table 3**.

**Reference:**

1. Kwon D, Belsky DW. A toolkit for quantification of biological age from blood chemistry and organ function test data: BioAge. *Geroscience.* 2021;43(6):2795-2808.

2. Levine ME, Lu AT, Quach A, et al. An epigenetic biomarker of aging for lifespan and healthspan. *Aging (Albany NY).* 2018;10(4):573-591.

3. Cassidy S, Chau JY, Catt M, Bauman A, Trenell MI. Cross-sectional study of diet, physical activity, television viewing and sleep duration in 233,110 adults from the UK Biobank; the behavioural phenotype of cardiovascular disease and type 2 diabetes. *BMJ Open.* 2016;6(3):e010038.

4. Li X, Zhou T, Ma H, et al. Healthy Sleep Patterns and Risk of Incident Arrhythmias. *J Am Coll Cardiol.* 2021;78(12):1197-1207.

5. Wang M, Zhou T, Li X, et al. Baseline Vitamin D Status, Sleep Patterns, and the Risk of Incident Type 2 Diabetes in Data From the UK Biobank Study. *Diabetes Care.* 2020;43(11):2776-2784.

6. Heath L, Jebb SA, Aveyard P, Piernas C. Obesity, metabolic risk and adherence to healthy lifestyle behaviours: prospective cohort study in the UK Biobank. *BMC Med.* 2022;20(1):65.

7. Kurki MI, Karjalainen J, Palta P, et al. FinnGen provides genetic insights from a well-phenotyped isolated population. *Nature.* 2023;613(7944):508-518.

**Supplementary Table 1.** Diagnosis codes or definitions used for outcomes and research variables in UK Biobank.

| **Variables** | **Field ID in UK Biobank** | **Code** |
| --- | --- | --- |
| Outcomes | | |
| Abdominal aortic aneurysm | 41270, 40001, and 40002  41272 | I71.3, I71.4  L18*, L19*, L254, L27*, L28*, L464 |
| Research variables | | |
| Telomere length | 22192 |  |
| Age | 21022 |  |
| Sex | 31 |  |
| Ethnicity | 21000 |  |
| Townsend deprivation index | 22189 |  |
| Education | 6138 |  |
| Body mass index | 21001 |  |
| C-reactive protein | 30710 |  |
| Glycated hemoglobin | 30750 |  |
| Low density lipoprotein | 30780 |  |
| Blood pressure | 4079-4080 |  |
| Drinking status | 20117 |  |
| Smoking status | 20116 |  |
| Metabolic Equivalent Task | 22040 |  |
| Hypertension | 6150, 131286-131295 | 4, I10-I13, I15 |
| Diabetes | 2443, 130706-130715  , | 1, E10-E14 |
| Hyperlipidemia | 130814-130815 | E78 |
| Antihypertensive drugs | 6177, 6153, 20003 | 2, 1140860696, 1140860728, 1140860738, 1140860750, 1140860752, 1140860764, 1140860790, 1140860806, 1140860882, 1140860904, 1140864952, 1140888552, 1140888556, 1140888560, 1140923712, 1141153328, 1141164148, 1141165470, 1141180592, 1140916356, 1141145660, 1141151016, 1141152998, 1141156836, 1141166006, 1141171336, 1141172682, 1141187788, 1141201038, 1140860426, 1140861088, 1140861190, 1140861276, 1140872568, 1140879802, 1140879810, 1140888646, 1140923572, 1140928226, 1141153026, 1141165470, 1140888510, 1141153328, 1140860332, 1140860404, 1140860422, 1140860562, 1140864950, 1140926778, 1140879806, 1140866244, 1140860308 |
| Antidiabetic drugs | 6177, 6153, 20003 | 3, 1140883066, 1140884600, 1141189090, 1140857494, 1140874646, 1140874650, 1140874658, 1140874718, 1140874744, 1141152590, 1141153254, 1141157284, 1141168660, 1141171646, 1141173882, 1141177600, 1140868902 |
| Cholesterol-lowering drugs | 6177, 6153, 20003 | 1, 1140861924, 1140861944, 1140861954, 1140862026, 1141157260, 1140861892, 1141192736, 1140861958, 1140888594, 1140888648, 1140910632, 1140910654, 1141146234, 1141192410 |

**Supplementary Table 2.** Details for healthy dietary or sleep scores in the UK Biobank.

| **Variables** | **Filed ID** | **Descriptions** | **Criteria for healthy diet** |
| --- | --- | --- | --- |
| vegetables | 1289 | Cooked vegetables | ≥4 tablespoons/day |
|  | 1299 | Salad/raw vegetables |  |
| Fruits | 1309 | Fresh fruits | ≥3 pieces/day |
|  | 1319 | Dried fruits |  |
| Fish | 1329 | Oily fish | ≥2 times/week |
|  | 1339 | Non-oily fish |  |
| Processed meats | 1349 | Processed meats | <2 times/week |
| Unprocessed red meats | 1369 | Beef | <2 times/week |
|  | 1379 | Lamb/mutton |  |
|  | 1389 | Pork |  |
| Sleep duration | 1160 | About how many hours sleep do you get in every 24 hours? | 7–8 hours per day |
| Sleep chronotype | 1180 | Do you consider yourself to be? and the possible responses included “Definitely a 'morning' person”, “More a 'morning' than 'evening' person”, “More an 'evening' than a 'morning' person”, “Definitely an 'evening' person”, “Do not know” and “Prefer not to answer” | Definitely a 'morning' person or more a 'morning' than 'evening' person |
| Insomnia | 1200 | Do you have trouble falling asleep at night or do you wake up in the middle of the night? | Never/rarely or sometimes |
| Snoring | 1210 | Does your partner or a close relative or friend complain about your snoring? | No |
| Daytime sleepiness | 1220 | How likely are you to doze off or fall asleep during the daytime when you don't mean to? (e.g. when working, reading or driving) | Never/rarely or sometimes |

**Supplementary Table 3.** Summary results of independent SNPs used for PRS construction.

| **Chromosome** | **Position** | **Effect allele** | **Other allele** | **rsID** | **OR** | ***P* value** |
| --- | --- | --- | --- | --- | --- | --- |
| 12 | 57133500 | C | T | rs11172113 | 0.872046 | 4.95E-08 |
| 12 | 18248945 | C | T | rs118156215 | 1.303222 | 1.16E-07 |
| 12 | 4493152 | C | T | rs11610054 | 0.705458 | 1.71E-07 |
| 19 | 43648948 | G | A | rs4760 | 1.170481 | 1.02E-06 |
| 9 | 87660127 | G | A | rs3128472 | 1.126937 | 1.33E-06 |
| 11 | 102931118 | G | A | rs12788925 | 1.123688 | 1.79E-06 |
| 12 | 121957871 | G | A | rs830124 | 0.86167 | 2.07E-06 |
| 5 | 75352778 | T | A | rs12654264 | 1.120265 | 3.30E-06 |
| 1 | 181496037 | A | G | rs12734881 | 1.222128 | 4.07E-06 |
| 6 | 32623176 | C | T | rs9271588 | 0.893456 | 4.59E-06 |
| 2 | 119425040 | A | G | rs74576273 | 0.647464 | 5.02E-06 |
| 20 | 56230648 | G | A | rs10485804 | 1.118157 | 5.76E-06 |
| 4 | 186430620 | G | A | rs6840417 | 1.116508 | 9.46E-06 |
| 1 | 109369429 | G | A | rs17646665 | 0.750811 | 1.26E-05 |
| 2 | 190323115 | A | C | rs1074247 | 0.71136 | 1.70E-05 |
| 12 | 132017451 | C | A | rs3923761 | 1.472141 | 1.86E-05 |
| 15 | 76538895 | A | G | rs2454450 | 0.894708 | 2.07E-05 |
| 1 | 171138879 | T | G | rs12143366 | 1.495997 | 2.29E-05 |
| 14 | 101467830 | A | G | rs117316297 | 0.639534 | 2.44E-05 |
| 17 | 62678385 | G | T | rs7350907 | 0.894108 | 2.63E-05 |
| 11 | 7542256 | A | G | rs12788646 | 1.135208 | 2.85E-05 |
| 3 | 170809750 | C | A | rs114177190 | 1.214493 | 3.16E-05 |
| 7 | 138188094 | C | G | rs73729501 | 1.325066 | 3.19E-05 |

Abbreviation: SNP, single nucleotide polymorphism; PRS, polygenic risk score; OR, odds ratio.

**Supplementary Table 4.** Baseline characteristics of study participants with or without abdominal aortic aneurysm.

| **Variables** | **Total** | **Abdominal aortic aneurysm** | | ***p* value** |
| --- | --- | --- | --- | --- |
|  |  | **No** | **Yes** |  |
| **N** | 311646 | 310307 | 1339 |  |
| **Age (years)** | 58.00 [50.00, 63.00] | 58.00 [50.00, 63.00] | 65.00 [61.00, 67.00] | <0.001 |
| **Women, n (%)** | 168249 (53.99) | 168044 (54.15) | 205 (15.31) | <0.001 |
| **White, n (%)** | 294574 (94.52) | 293266 (94.51) | 1308 (97.68) | <0.001 |
| **TDI** | -2.20 [-3.67, 0.39] | -2.20 [-3.67, 0.39] | -1.88 [-3.56, 0.79] | 0.005 |
| **Education, n (%)** |  |  |  | <0.001 |
| College | 101091 (32.44) | 100868 (32.51) | 223 (16.65) |  |
| High school | 35071 (11.25) | 34962 (11.27) | 109 (8.14) |  |
| Middle school | 67420 (21.63) | 67131 (21.63) | 289 (21.58) |  |
| Others | 108064 (34.68) | 107346 (34.59) | 718 (53.62) |  |
| **BMI, Kg/m^2^** | 26.68 [24.11, 29.79] | 26.67 [24.10, 29.78] | 28.01 [25.63, 30.84] | <0.001 |
| **CRP, mg/L** | 1.30 [0.64, 2.67] | 1.29 [0.64, 2.66] | 2.15 [1.08, 4.40] | <0.001 |
| **Drinking status, n (%)** |  |  |  | <0.001 |
| Never | 13303 (4.27) | 13271 (4.28) | 32 (2.39) |  |
| Previous | 10545 (3.38) | 10476 (3.38) | 69 (5.15) |  |
| Current | 287103 (92.12) | 285868 (92.12) | 1235 (92.23) |  |
| **Smoking status, n (%)** |  |  |  | <0.001 |
| Never | 171217 (54.94) | 170969 (55.10) | 248 (18.52) |  |
| Previous | 107241 (34.41) | 106583 (34.35) | 658 (49.14) |  |
| Current | 31733 (10.18) | 31310 (10.09) | 423 (31.59) |  |
| **Physical activity, n (%)** |  |  |  | 0.014 |
| Regular | 122958 (39.45) | 122479 (39.47) | 479 (35.77) |  |
| Excessive | 75514 (24.23) | 75179 (24.23) | 335 (25.02) |  |
| Poor | 43693 (14.02) | 43473 (14.01) | 220 (16.43) |  |
| **Sleep pattern, n (%)** |  |  |  | <0.001 |
| Healthy | 151320 (48.56) | 150764 (48.59) | 556 (41.52) |  |
| Intermediate | 100598 (32.28) | 100075 (32.25) | 523 (39.06) |  |
| Poor | 5807 (1.86) | 5764 (1.86) | 43 (3.21) |  |
| **Diet pattern, n (%)** |  |  |  | <0.001 |
| Healthy | 117408 (37.67) | 117027 (37.71) | 381 (28.45) |  |
| Intermediate | 145882 (46.81) | 145198 (46.79) | 684 (51.08) |  |
| Poor | 35506 (11.39) | 35290 (11.37) | 216 (16.13) |  |
| **Medical history, n (%)** |  |  |  |  |
| Hyperlipidemia | 144832 (46.47) | 143837 (46.35) | 995 (74.31) | <0.001 |
| Hypertension | 170827 (54.81) | 169723 (54.70) | 1104 (82.45) | <0.001 |
| Diabetes mellitus | 17765 (5.70) | 17627 (5.68) | 138 (10.31) | <0.001 |

Abbreviation: TDI, Townsend deprivation index; CRP, C-reactive protein; BMI, body mass index.

**Supplementary Table 5**. Baseline characteristics of study participants grouped by KDM-BA acceleration quartiles.

| **Variables** | **Total** | **KDM-BA** **acceleration** | | | | ***p* for overall** | ***p* for trend** |
| --- | --- | --- | --- | --- | --- | --- | --- |
|  |  | **Q1** | **Q2** | **Q3** | **Q4** |  |  |
| **N** | 311646 | 77912 | 77911 | 77911 | 77912 |  |  |
| **Age (years)** | 58.00 [50.00, 63.00] | 58.00 [50.00, 63.00] | 57.00 [49.00, 63.00] | 57.00 [50.00, 63.00] | 58.00 [51.00, 63.00] | <0.001 | <0.001 |
| **Women, n (%)** | 168249 (53.99) | 15 (0.02) | 31672 (40.65) | 69154 (88.76) | 67408 (86.52) | <0.001 | <0.001 |
| **White, n (%)** | 294574 (94.52) | 75229 (96.56) | 73977 (94.95) | 74333 (95.41) | 71035 (91.17) | <0.001 | <0.001 |
| **TDI** | -2.20 [-3.67, 0.39] | -2.43 [-3.80, -0.09] | -2.18 [-3.65, 0.41] | -2.24 [-3.69, 0.25] | -1.88 [-3.49, 1.00] | <0.001 | <0.001 |
| **Education, n (%)** |  |  |  |  |  | <0.001 | <0.001 |
| College | 101091 (32.44) | 30869 (39.62) | 26688 (34.25) | 24906 (31.97) | 18628 (23.91) |  |  |
| High school | 35071 (11.25) | 8527 (10.94) | 8768 (11.25) | 9523 (12.22) | 8253 (10.59) |  |  |
| Middle school | 67420 (21.63) | 14463 (18.56) | 16100 (20.66) | 18313 (23.51) | 18544 (23.80) |  |  |
| Others | 108064 (34.68) | 24053 (30.87) | 26355 (33.83) | 25169 (32.30) | 32487 (41.70) |  |  |
| **BMI, Kg/m^2^** | 26.68 [24.11, 29.79] | 26.36 [24.30, 28.69] | 26.54 [23.77, 29.55] | 25.94 [23.43, 29.19] | 28.13 [25.08, 31.97] | <0.001 | <0.001 |
| **CRP, mg/L** | 1.30 [0.64, 2.67] | 0.76 [0.45, 1.27] | 1.36 [0.67, 2.49] | 1.31 [0.65, 2.67] | 2.51 [1.23, 5.06] | <0.001 | <0.001 |
| **Drinking status, n (%)** |  |  |  |  |  | <0.001 | <0.001 |
| Never | 13303 (4.27) | 1595 (2.05) | 2613 (3.35) | 3607 (4.63) | 5488 (7.04) |  |  |
| Previous | 10545 (3.38) | 2261 (2.90) | 2565 (3.29) | 2592 (3.33) | 3127 (4.01) |  |  |
| Current | 287103 (92.12) | 73939 (94.90) | 72573 (93.15) | 71559 (91.85) | 69032 (88.60) |  |  |
| **Smoking status, n (%)** |  |  |  |  |  | <0.001 | <0.001 |
| Never | 171217 (54.94) | 41828 (53.69) | 40523 (52.01) | 45163 (57.97) | 43703 (56.09) |  |  |
| Previous | 107241 (34.41) | 28815 (36.98) | 28518 (36.60) | 25285 (32.45) | 24623 (31.60) |  |  |
| Current | 31733 (10.18) | 6980 (8.96) | 8524 (10.94) | 7116 (9.13) | 9113 (11.70) |  |  |
| **Physical activity, n (%)** |  |  |  |  |  | <0.001 | <0.001 |
| Regular | 122958 (39.45) | 33484 (42.98) | 31458 (40.38) | 30561 (39.23) | 27455 (35.24) |  |  |
| Excessive | 75514 (24.23) | 21565 (27.68) | 19751 (25.35) | 18098 (23.23) | 16100 (20.66) |  |  |
| Poor | 43693 (14.02) | 10532 (13.52) | 11042 (14.17) | 10463 (13.43) | 11656 (14.96) |  |  |
| **Sleep pattern, n (%)** |  |  |  |  |  | <0.001 | 0.001 |
| Healthy | 151320 (48.56) | 37779 (48.49) | 37868 (48.60) | 40128 (51.50) | 35545 (45.62) |  |  |
| Intermediate | 100598 (32.28) | 25033 (32.13) | 25153 (32.28) | 23848 (30.61) | 26564 (34.09) |  |  |
| Poor | 5807 (1.86) | 1198 (1.54) | 1418 (1.82) | 1241 (1.59) | 1950 (2.50) |  |  |
| **Diet pattern, n (%)** |  |  |  |  |  | <0.001 | <0.001 |
| Healthy | 117408 (37.67) | 24878 (31.93) | 28108 (36.08) | 34285 (44.01) | 30137 (38.68) |  |  |
| Intermediate | 145882 (46.81) | 39157 (50.26) | 36540 (46.90) | 34511 (44.30) | 35674 (45.79) |  |  |
| Poor | 35506 (11.39) | 11296 (14.50) | 10022 (12.86) | 6280 (8.06) | 7908 (10.15) |  |  |
| **Medical history, n (%)** |  |  |  |  |  |  |  |
| Hyperlipidemia | 144832 (46.47) | 35585 (45.67) | 32680 (41.95) | 32018 (41.10) | 44549 (57.18) | <0.001 | <0.001 |
| Hypertension | 170827 (54.81) | 40168 (51.56) | 39554 (50.77) | 34662 (44.49) | 56443 (72.44) | <0.001 | <0.001 |
| Diabetes mellitus | 17765 (5.70) | 3817 (4.90) | 4771 (6.12) | 3145 (4.04) | 6032 (7.74) | <0.001 | <0.001 |

Abbreviation: KDM-BA, Klemera-Doubal method biological age; TDI, Townsend deprivation index; CRP, C-reactive protein; BMI, body mass index. Note: Q1-Q4 (quartiles) of KDM-BA acceleration: Q1 (< -0.68), Q2 (< 0.35, ≥ -0.68), Q3 (< 0.72, ≥ 0.35), Q4 (≥ 0.72).

**Supplementary Table 6**. Baseline characteristics of study participants grouped by PhenoAge acceleration quartiles.

| **Variables** | **Total** | **PhenoAge acceleration** | | | | ***p* for overall** | ***p* for trend** |
| --- | --- | --- | --- | --- | --- | --- | --- |
|  |  | **Q1** | **Q2** | **Q3** | **Q4** |  |  |
| **N** | 311646 | 77912 | 77911 | 77911 | 77912 |  |  |
| **Age (years)** | 58.00 [50.00, 63.00] | 58.00 [50.00, 63.00] | 58.00 [50.00, 63.00] | 58.00 [50.00, 63.00] | 58.00 [50.00, 63.00] | 0.141 | 0.075 |
| **Women, n (%)** | 168249 (53.99) | 51030 (65.50) | 42017 (53.93) | 38326 (49.19) | 36876 (47.33) | <0.001 | <0.001 |
| **White, n (%)** | 294574 (94.52) | 74535 (95.67) | 74211 (95.25) | 73630 (94.51) | 72198 (92.67) | <0.001 | <0.001 |
| **TDI** | -2.20 [-3.67, 0.39] | -2.46 [-3.82, -0.22] | -2.35 [-3.73, 0.03] | -2.21 [-3.67, 0.39] | -1.66 [-3.38, 1.35] | <0.001 | <0.001 |
| **Education, n (%)** |  |  |  |  |  | <0.001 | <0.001 |
| College | 101091 (32.44) | 29734 (38.16) | 26875 (34.49) | 24314 (31.21) | 20168 (25.89) |  |  |
| High school | 35071 (11.25) | 9208 (11.82) | 8929 (11.46) | 8733 (11.21) | 8201 (10.53) |  |  |
| Middle school | 67420 (21.63) | 16512 (21.19) | 16776 (21.53) | 17074 (21.91) | 17058 (21.89) |  |  |
| Others | 108064 (34.68) | 22458 (28.82) | 25331 (32.51) | 27790 (35.67) | 32485 (41.69) |  |  |
| **BMI, Kg/m^2^** | 26.68 [24.11, 29.79] | 24.88 [22.79, 27.32] | 26.32 [24.02, 29.00] | 27.34 [24.81, 30.32] | 28.64 [25.62, 32.46] | <0.001 | <0.001 |
| **CRP, mg/L** | 1.30 [0.64, 2.67] | 0.57 [0.34, 0.98] | 1.05 [0.62, 1.78] | 1.65 [0.95, 2.85] | 3.26 [1.71, 6.22] | <0.001 | <0.001 |
| **Drinking status, n (%)** |  |  |  |  |  | <0.001 | <0.001 |
| Never | 13303 (4.27) | 2841 (3.65) | 2954 (3.79) | 3343 (4.29) | 4165 (5.35) |  |  |
| Previous | 10545 (3.38) | 2219 (2.85) | 2223 (2.85) | 2533 (3.25) | 3570 (4.58) |  |  |
| Current | 287103 (92.12) | 72712 (93.33) | 72616 (93.20) | 71865 (92.24) | 69910 (89.73) |  |  |
| **Smoking status, n (%)** |  |  |  |  |  | <0.001 | <0.001 |
| Never | 171217 (54.94) | 47137 (60.50) | 45036 (57.80) | 42215 (54.18) | 36829 (47.27) |  |  |
| Previous | 107241 (34.41) | 26541 (34.07) | 26892 (34.52) | 27273 (35.01) | 26535 (34.06) |  |  |
| Current | 31733 (10.18) | 3933 (5.05) | 5658 (7.26) | 8049 (10.33) | 14093 (18.09) |  |  |
| **Physical activity, n (%)** |  |  |  |  |  | <0.001 | <0.001 |
| Regular | 122958 (39.45) | 32442 (41.64) | 31600 (40.56) | 30778 (39.50) | 28138 (36.12) |  |  |
| Excessive | 75514 (24.23) | 20355 (26.13) | 19732 (25.33) | 18751 (24.07) | 16676 (21.40) |  |  |
| Poor | 43693 (14.02) | 9174 (11.77) | 10158 (13.04) | 10918 (14.01) | 13443 (17.25) |  |  |
| **Sleep pattern, n (%)** |  |  |  |  |  | <0.001 | <0.001 |
| Healthy | 151320 (48.56) | 41431 (53.18) | 39349 (50.51) | 37212 (47.76) | 33328 (42.78) |  |  |
| Intermediate | 100598 (32.28) | 22692 (29.13) | 24196 (31.06) | 25776 (33.08) | 27934 (35.85) |  |  |
| Poor | 5807 (1.86) | 1013 (1.30) | 1168 (1.50) | 1461 (1.88) | 2165 (2.78) |  |  |
| **Diet pattern, n (%)** |  |  |  |  |  | <0.001 | <0.001 |
| Healthy | 117408 (37.67) | 36305 (46.60) | 30637 (39.32) | 27280 (35.01) | 23186 (29.76) |  |  |
| Intermediate | 145882 (46.81) | 33317 (42.76) | 36474 (46.81) | 37539 (48.18) | 38552 (49.48) |  |  |
| Poor | 35506 (11.39) | 5898 (7.57) | 8072 (10.36) | 9721 (12.48) | 11815 (15.16) |  |  |
| **Medical history, n (%)** |  |  |  |  |  |  |  |
| Hyperlipidemia | 144832 (46.47) | 34041 (43.69) | 35832 (45.99) | 36823 (47.26) | 38136 (48.95) | <0.001 | <0.001 |
| Hypertension | 170827 (54.81) | 35999 (46.20) | 40835 (52.41) | 44471 (57.08) | 49522 (63.56) | <0.001 | <0.001 |
| Diabetes mellitus | 17765 (5.70) | 1682 (2.16) | 2587 (3.32) | 4096 (5.26) | 9400 (12.06) | <0.001 | <0.001 |

Abbreviation: PhenoAge, phenotypic age; TDI, Townsend deprivation index; CRP, C-reactive protein; BMI, body mass index. Note: Q1-Q4 (quartiles) of PhenoAge acceleration: Q1 (< -0.69), Q2 (< -0.08, ≥ -0.69), Q3 (< 0.60, ≥ -0.08), Q4 (≥ 0.60).

**Supplementary Table 7.** Association of PRS for AAA with incident AAA using Cox proportional hazards regression.

| **Variables** | **Cases/N** | **Incidence rate^∗^** | **Model 1** | |  | **Model 2** | |
| --- | --- | --- | --- | --- | --- | --- | --- |
|  |  |  | **HR (95%CI)** | ***p* value** |  | **HR (95%CI)** | ***p* value** |
| **PRS, continuous** | 1191/279750 | 3.462 | 1.15 (1.08, 1.22) | <0.001 |  | 1.15 (1.08, 1.22) | <0.001 |
| **PRS, categorical** |  |  |  |  |  |  |  |
| Low | 196/55960 | 2.848 | 1 (reference) |  |  | 1 (reference) |  |
| Intermediate | 709/168607 | 3.42 | 1.19 (1.01, 1.39) | 0.034 |  | 1.19 (1.02, 1.40) | 0.028 |
| High | 286/55183 | 4.215 | 1.48 (1.23, 1.77) | <0.001 |  | 1.48 (1.23, 1.78) | <0.001 |

Adjusted for age, sex, ethnicity, Townsend deprivation index, education levels, body mass index, C-reactive protein, smoking and drinking status, physical activity, sleep and dietary patterns, history of hyperlipemia, hypertension, diabetes mellitus, genotyping batch, and the first 10 genetic principal components.

Abbreviation: PRS, polygenic risk score; AAA, abdominal aortic aneurysm; HR, hazard ratio; CI, confidence interval.

**Supplementary Table 8.** Association between biological ageing and the risk of abdominal aortic aneurysm after additionally adjusted for genetic susceptibility.

|  | **Cases/N** | **Incidence rate^∗^** | **Model 1+PRS** | |  | **Model 2+PRS** | |
| --- | --- | --- | --- | --- | --- | --- | --- |
|  |  |  | **HR (95%CI)** | ***p* value** |  | **HR (95%CI)** | ***p* value** |
| **Telomere length** |  |  |  |  |  |  |  |
| Q1 | 482/69939 | 5.661 | 1 (reference) |  |  | 1 (reference) |  |
| Q2 | 316/69936 | 3.676 | 0.82 (0.71, 0.95) | 0.006 |  | 0.85 (0.73, 0.98) | 0.022 |
| Q3 | 242/69937 | 2.804 | 0.76 (0.65, 0.89) | 0.001 |  | 0.80 (0.69, 0.94) | 0.005 |
| Q4 | 151/69938 | 1.744 | 0.62 (0.51, 0.74) | <0.001 |  | 0.66 (0.55, 0.79) | <0.001 |
| Continuous | 1191/279750 | 3.462 | 0.82 (0.78, 0.87) | <0.001 |  | 0.84 (0.79, 0.89) | <0.001 |
| **KDM-BA acceleration** |  |  |  |  |  |  |  |
| Q1 | 365/69938 | 4.242 | 1 (reference) |  |  | 1 (reference) |  |
| Q2 | 418/69937 | 4.87 | 1.63 (1.41, 1.88) | <0.001 |  | 1.25 (1.08, 1.45) | 0.003 |
| Q3 | 143/69937 | 1.653 | 1.95 (1.59, 2.40) | <0.001 |  | 1.25 (1.01, 1.55) | 0.043 |
| Q4 | 265/69938 | 3.095 | 3.27 (2.75, 3.90) | <0.001 |  | 1.71 (1.39, 2.11) | <0.001 |
| Continuous | 1191/279750 | 3.462 | 1.53 (1.44, 1.63) | <0.001 |  | 1.21 (1.13, 1.30) | <0.001 |
| **PhenoAge acceleration** |  |  |  |  |  |  |  |
| Q1 | 117/69938 | 1.341 | 1 (reference) |  |  | 1 (reference) |  |
| Q2 | 189/69937 | 2.179 | 1.27 (1.01, 1.60) | 0.040 |  | 1.15 (0.91, 1.45) | 0.244 |
| Q3 | 293/69937 | 3.403 | 1.74 (1.41, 2.16) | <0.001 |  | 1.43 (1.15, 1.77) | 0.001 |
| Q4 | 592/69938 | 7.055 | 3.26 (2.67, 3.98) | <0.001 |  | 2.08 (1.68, 2.57) | <0.001 |
| Continuous | 1191/279750 | 3.462 | 1.60 (1.52, 1.69) | <0.001 |  | 1.37 (1.28, 1.46) | <0.001 |

^*^The incidence rate was reported as per 10,000 person-years.

Model 1 adjusted for age, sex, ethnicity, Townsend deprivation index, education levels.

Model 2 adjusted for model 1 plus body mass index, C-reactive protein, smoking and drinking status, physical activity, sleep and dietary patterns, history of hyperlipemia, hypertension, diabetes mellitus.

Abbreviation: KDM-BA, Klemera-Doubal method biological age; PhenoAge, phenotypic age; HR, hazard ratio; CI, confidence interval.

**Supplementary Table 9.** Subgroup analysis for the association between KDM-BA acceleration with the risk of abdominal aortic aneurysm.

| **Subgroup** | **Cases/N** | **KDM-BA acceleration** | | | | ***p* for interaction** |
| --- | --- | --- | --- | --- | --- | --- |
|  |  | **Q1** | **Q2** | **Q3** | **Q4** |  |
| **Age** |  |  |  |  |  | 0.931 |
| <45 | 5/32843 | 1 (reference) | 2.88 (0.48, 17.25) | 1.75 (0.19, 15.77) | 1.06 (0.12, 9.48) |  |
| ≥45, <55 | 60/89533 | 1 (reference) | 1.24 (0.62, 2.47) | 0.96 (0.36, 2.56) | 1.10 (0.43, 2.84) |  |
| ≥55, <65 | 600/131278 | 1 (reference) | 1.16 (0.95, 1.43) | 1.20 (0.88, 1.64) | 1.65 (1.23, 2.21) |  |
| ≥65 | 674/57992 | 1 (reference) | 1.28 (1.06, 1.55) | 1.30 (0.98, 1.73) | 1.72 (1.31, 2.27) |  |
| **Age** |  |  |  |  |  | 0.522 |
| <65 | 665/253654 | 1 (reference) | 1.08 (0.89, 1.32) | 1.16 (0.87, 1.54) | 1.36 (1.03, 1.79) |  |
| ≥65 | 674/57992 | 1 (reference) | 1.28 (1.06, 1.55) | 1.30 (0.98, 1.73) | 1.72 (1.31, 2.27) |  |
| **Sex** |  |  |  |  |  | 0.250 |
| Women | 205/168249 | 1 (reference) | 0.54 (0.32, 0.89) | 0.77 (0.49, 1.20) | 1.10 (0.71, 1.69) |  |
| Men | 1134/143397 | 1 (reference) | 1.05 (0.85, 1.29) | 1.26 (1.03, 1.54) | 1.53 (1.25, 1.88) |  |
| **Ethnicity** |  |  |  |  |  | 0.398 |
| White | 1308/294574 | 1 (reference) | 1.25 (1.08, 1.43) | 1.30 (1.06, 1.59) | 1.72 (1.42, 2.10) |  |
| Non-White | 31/17072 | 1 (reference) | 1.59 (0.60, 4.24) | 3.58 (1.15, 11.14) | 2.76 (0.77, 9.94) |  |
| **TDI** |  |  |  |  |  | 0.780 |
| Low | 411/104004 | 1 (reference) | 1.47 (1.15, 1.88) | 1.46 (1.00, 2.12) | 1.64 (1.12, 2.39) |  |
| Moderate | 445/103925 | 1 (reference) | 1.09 (0.86, 1.38) | 1.44 (1.02, 2.03) | 1.98 (1.42, 2.76) |  |
| High | 483/103717 | 1 (reference) | 1.22 (0.97, 1.55) | 1.06 (0.76, 1.48) | 1.57 (1.16, 2.14) |  |
| **Education** |  |  |  |  |  | 0.520 |
| College | 223/101091 | 1 (reference) | 1.37 (0.98, 1.93) | 1.66 (1.00, 2.76) | 2.45 (1.48, 4.06) |  |
| High school | 109/35071 | 1 (reference) | 1.07 (0.65, 1.76) | 1.77 (0.93, 3.36) | 1.42 (0.73, 2.79) |  |
| Middle school | 289/67420 | 1 (reference) | 1.24 (0.92, 1.66) | 1.61 (1.04, 2.49) | 2.05 (1.37, 3.08) |  |
| Others | 718/108064 | 1 (reference) | 1.20 (0.99, 1.44) | 1.19 (0.91, 1.55) | 1.46 (1.12, 1.89) |  |
| **Hypertension** |  |  |  |  |  | 0.097 |
| No | 235/140819 | 1 (reference) | 1.71 (1.24, 2.36) | 1.55 (0.89, 2.72) | 2.00 (1.17, 3.40) |  |
| Yes | 1104/170827 | 1 (reference) | 1.15 (0.99, 1.33) | 1.34 (1.08, 1.66) | 1.65 (1.33, 2.04) |  |
| **Hyperlipidemia** |  |  |  |  |  | 0.978 |
| No | 344/166814 | 1 (reference) | 1.28 (0.97, 1.68) | 1.26 (0.83, 1.92) | 1.83 (1.25, 2.67) |  |
| Yes | 995/144832 | 1 (reference) | 1.23 (1.05, 1.43) | 1.20 (0.95, 1.52) | 1.67 (1.32, 2.10) |  |
| **Diabetes** |  |  |  |  |  | 0.283 |
| No | 1201/293881 | 1 (reference) | 1.20 (1.04, 1.39) | 1.26 (1.01, 1.56) | 1.75 (1.42, 2.15) |  |
| Yes | 138/17765 | 1 (reference) | 1.33 (0.88, 2.01) | 0.94 (0.51, 1.73) | 1.26 (0.67, 2.35) |  |

Adjusted for age, sex, ethnicity, TDI, education levels body mass index, C-reactive protein, smoking and drinking status, physical activity, sleep and diet patterns, history of hyperlipemia, hypertension, and diabetes mellitus.

Abbreviation: KDM-BA, Klemera-Doubal method biological age; TDI, Townsend deprivation index.

**Supplementary Table 10.** Subgroup analysis for the association between PhenoAge acceleration with the risk of abdominal aortic aneurysm.

| **Subgroup** | **Cases/N** | **PhenoAge acceleration** | | | | ***p* for interaction** |
| --- | --- | --- | --- | --- | --- | --- |
|  |  | **Q1** | **Q2** | **Q3** | **Q4** |  |
| **Age** |  |  |  |  |  | 0.880 |
| <45 | 5/32843 | 1 (reference) | 1.27 (0.00, Inf) | 264894934.25 (44042020.71, 1593235847.44) | 595843500.50 (99066265.35, 3583757556.85) |  |
| ≥45, <55 | 60/89533 | 1 (reference) | 2.17 (0.69, 6.86) | 2.62 (0.86, 7.97) | 3.28 (1.08, 9.99) |  |
| ≥55, <65 | 600/131278 | 1 (reference) | 1.09 (0.78, 1.51) | 1.32 (0.97, 1.80) | 2.28 (1.69, 3.07) |  |
| ≥65 | 674/57992 | 1 (reference) | 1.03 (0.77, 1.38) | 1.42 (1.08, 1.87) | 1.89 (1.44, 2.48) |  |
| **Age** |  |  |  |  |  | 0.979 |
| <65 | 665/253654 | 1 (reference) | 1.23 (0.90, 1.67) | 1.52 (1.13, 2.04) | 2.39 (1.79, 3.18) |  |
| ≥65 | 674/57992 | 1 (reference) | 1.03 (0.77, 1.38) | 1.42 (1.08, 1.87) | 1.89 (1.44, 2.48) |  |
| **Sex** |  |  |  |  |  | 0.292 |
| Women | 205/168249 | 1 (reference) | 0.72 (0.43, 1.22) | 1.16 (0.73, 1.84) | 2.10 (1.34, 3.28) |  |
| Men | 1134/143397 | 1 (reference) | 1.14 (0.91, 1.42) | 1.55 (1.26, 1.90) | 2.05 (1.67, 2.51) |  |
| **Ethnicity** |  |  |  |  |  | 0.087 |
| White | 1308/294574 | 1 (reference) | 1.14 (0.91, 1.42) | 1.45 (1.17, 1.78) | 2.08 (1.70, 2.55) |  |
| Non-White | 31/17072 | 1 (reference) | 0.89 (0.18, 4.46) | 2.48 (0.63, 9.67) | 6.79 (1.80, 25.59) |  |
| **TDI** |  |  |  |  |  | 0.340 |
| Low | 411/104004 | 1 (reference) | 0.85 (0.59, 1.23) | 1.14 (0.81, 1.61) | 1.58 (1.13, 2.19) |  |
| Moderate | 445/103925 | 1 (reference) | 1.48 (0.99, 2.20) | 1.76 (1.20, 2.59) | 2.95 (2.03, 4.29) |  |
| High | 483/103717 | 1 (reference) | 1.23 (0.85, 1.79) | 1.50 (1.05, 2.13) | 2.18 (1.54, 3.08) |  |
| **Education** |  |  |  |  |  | 0.783 |
| College | 223/101091 | 1 (reference) | 0.85 (0.52, 1.38) | 1.22 (0.78, 1.90) | 1.65 (1.07, 2.56) |  |
| High school | 109/35071 | 1 (reference) | 1.14 (0.55, 2.36) | 1.05 (0.51, 2.15) | 1.92 (0.97, 3.78) |  |
| Middle school | 289/67420 | 1 (reference) | 1.28 (0.79, 2.07) | 1.89 (1.21, 2.96) | 2.22 (1.42, 3.48) |  |
| Others | 718/108064 | 1 (reference) | 1.05 (0.77, 1.41) | 1.69 (1.29, 2.22) | 2.26 (1.72, 2.97) |  |
| **Hypertension** |  |  |  |  |  | 0.464 |
| No | 235/140819 | 1 (reference) | 1.14 (0.66, 1.96) | 1.66 (1.01, 2.74) | 2.43 (1.49, 3.95) |  |
| Yes | 1104/170827 | 1 (reference) | 1.06 (0.85, 1.33) | 1.46 (1.19, 1.81) | 2.05 (1.66, 2.52) |  |
| **Hyperlipidemia** |  |  |  |  |  | 0.976 |
| No | 344/166814 | 1 (reference) | 1.10 (0.71, 1.69) | 1.41 (0.94, 2.12) | 2.01 (1.35, 2.99) |  |
| Yes | 995/144832 | 1 (reference) | 1.08 (0.84, 1.38) | 1.44 (1.15, 1.82) | 2.10 (1.68, 2.63) |  |
| **Diabetes** |  |  |  |  |  | 0.797 |
| No | 1201/293881 | 1 (reference) | 1.10 (0.88, 1.38) | 1.37 (1.11, 1.70) | 1.99 (1.61, 2.45) |  |
| Yes | 138/17765 | 1 (reference) | 1.62 (0.89, 2.96) | 2.78 (1.58, 4.87) | 2.61 (1.41, 4.82) |  |

Adjusted for age, sex, ethnicity, TDI, education levels body mass index, C-reactive protein, smoking and drinking status, physical activity, sleep and diet patterns, history of hyperlipemia, hypertension, and diabetes mellitus.

Abbreviation: PhenoAge, phenotypic age; TDI, Townsend deprivation index.

**Supplementary Table 11.** Sensitivity analysis for the associations between biological ageing and the risk of AAA after excluding participants diagnosed AAA within the first years of follow-up.

|  | **Cases/N** | **Incidence rate^∗^** | **Model 1** | |  | **Model 2** | |
| --- | --- | --- | --- | --- | --- | --- | --- |
|  |  |  | **HR (95%CI)** | ***p* value** |  | **HR (95%CI)** | ***p* value** |
| **Telomere length** |  |  |  |  |  |  |  |
| Q1 | 526/77900 | 5.554 | 1 (reference) |  |  | 1 (reference) |  |
| Q2 | 344/77900 | 3.598 | 0.82 (0.72, 0.94) | 0.004 |  | 0.84 (0.74, 0.97) | 0.015 |
| Q3 | 261/77899 | 2.719 | 0.75 (0.65, 0.88) | <0.001 |  | 0.79 (0.68, 0.92) | 0.002 |
| Q4 | 161/77900 | 1.672 | 0.61 (0.51, 0.73) | <0.001 |  | 0.65 (0.54, 0.78) | <0.001 |
| Continuous | 1292/311599 | 3.377 | 0.82 (0.77, 0.86) | <0.001 |  | 0.83 (0.79, 0.88) | <0.001 |
| **KDM-BA acceleration** |  |  |  |  |  |  |  |
| Q1 | 408/77900 | 4.261 | 1 (reference) |  |  | 1 (reference) |  |
| Q2 | 441/77900 | 4.617 | 1.60 (1.40, 1.84) | <0.001 |  | 1.23 (1.07, 1.41) | 0.004 |
| Q3 | 161/77899 | 1.673 | 1.92 (1.57, 2.34) | <0.001 |  | 1.24 (1.01, 1.52) | 0.043 |
| Q4 | 282/77900 | 2.964 | 3.06 (2.58, 3.62) | <0.001 |  | 1.60 (1.31, 1.95) | <0.001 |
| Continuous | 1292/311599 | 3.377 | 1.51 (1.42, 1.60) | <0.001 |  | 1.19 (1.11, 1.28) | <0.001 |
| **PhenoAge acceleration** |  |  |  |  |  |  |  |
| Q1 | 129/77900 | 1.328 | 1 (reference) |  |  | 1 (reference) |  |
| Q2 | 206/77900 | 2.135 | 1.26 (1.01, 1.57) | 0.039 |  | 1.14 (0.91, 1.42) | 0.255 |
| Q3 | 325/77899 | 3.392 | 1.78 (1.45, 2.19) | <0.001 |  | 1.46 (1.18, 1.79) | <0.001 |
| Q4 | 632/77900 | 6.777 | 3.26 (2.69, 3.95) | <0.001 |  | 2.09 (1.71, 2.57) | <0.001 |
| Continuous | 1292/311599 | 3.377 | 1.60 (1.53, 1.69) | <0.001 |  | 1.39 (1.31, 1.48) | <0.001 |

^*^The incidence rate was reported as per 10,000 person-years.

Model 1 adjusted for age, sex, ethnicity, Townsend deprivation index, education levels.

Model 2 adjusted for model 1 plus body mass index, C-reactive protein, smoking and drinking status, physical activity, sleep and dietary patterns, history of hyperlipemia, hypertension, diabetes mellitus.

Abbreviation: KDM-BA, Klemera-Doubal method biological age; PhenoAge, phenotypic age; HR, hazard ratio; CI, confidence interva

**Supplementary Fig. 1**. Flow chart of study participants


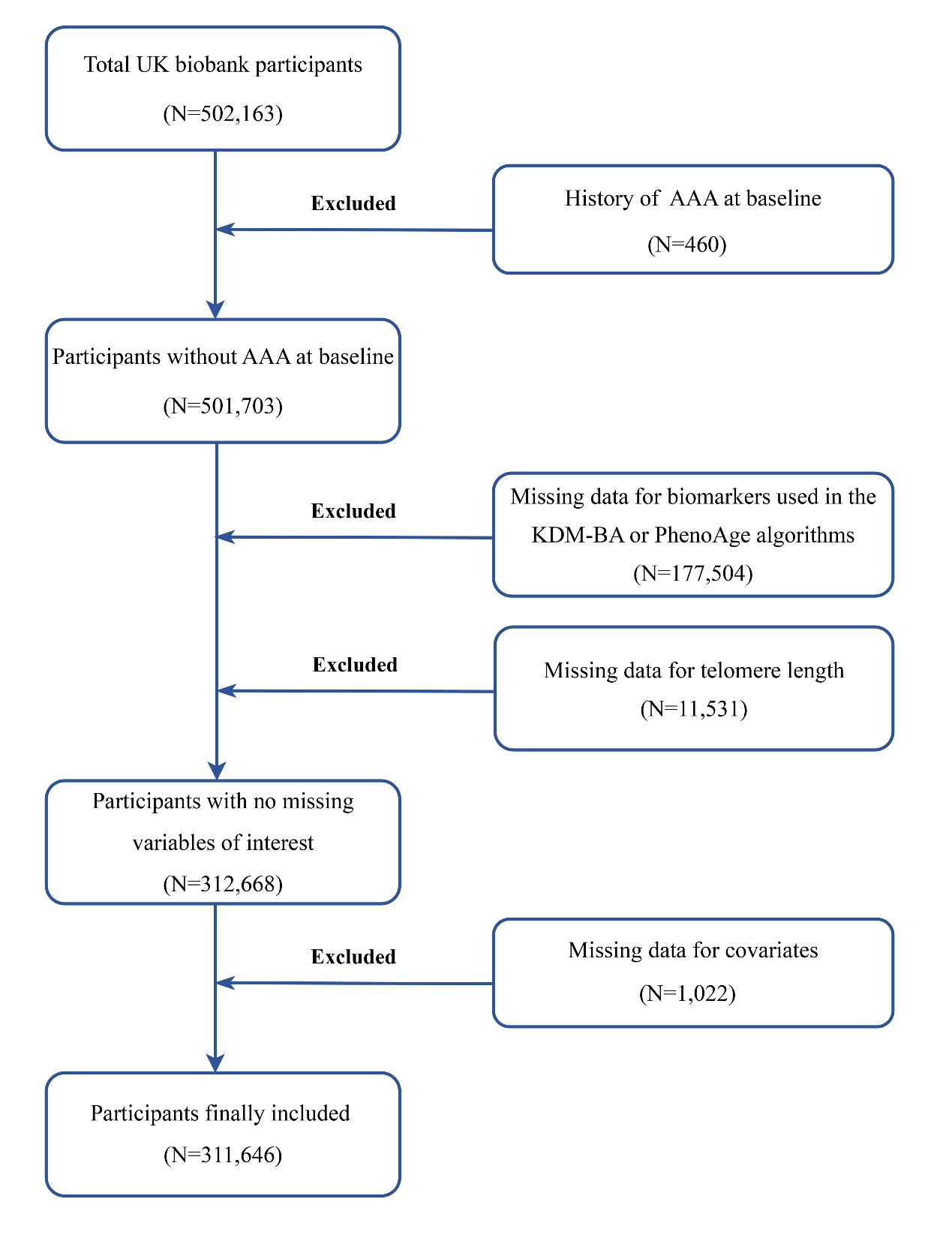


Abbreviation: AAA, abdominal aortic aneurysm; KDM-BA, Klemera-Doubal method biological age; PhenoAge, phenotypic age.

**Supplementary Fig. 2**. Correlations between biological age indicators and chronological age


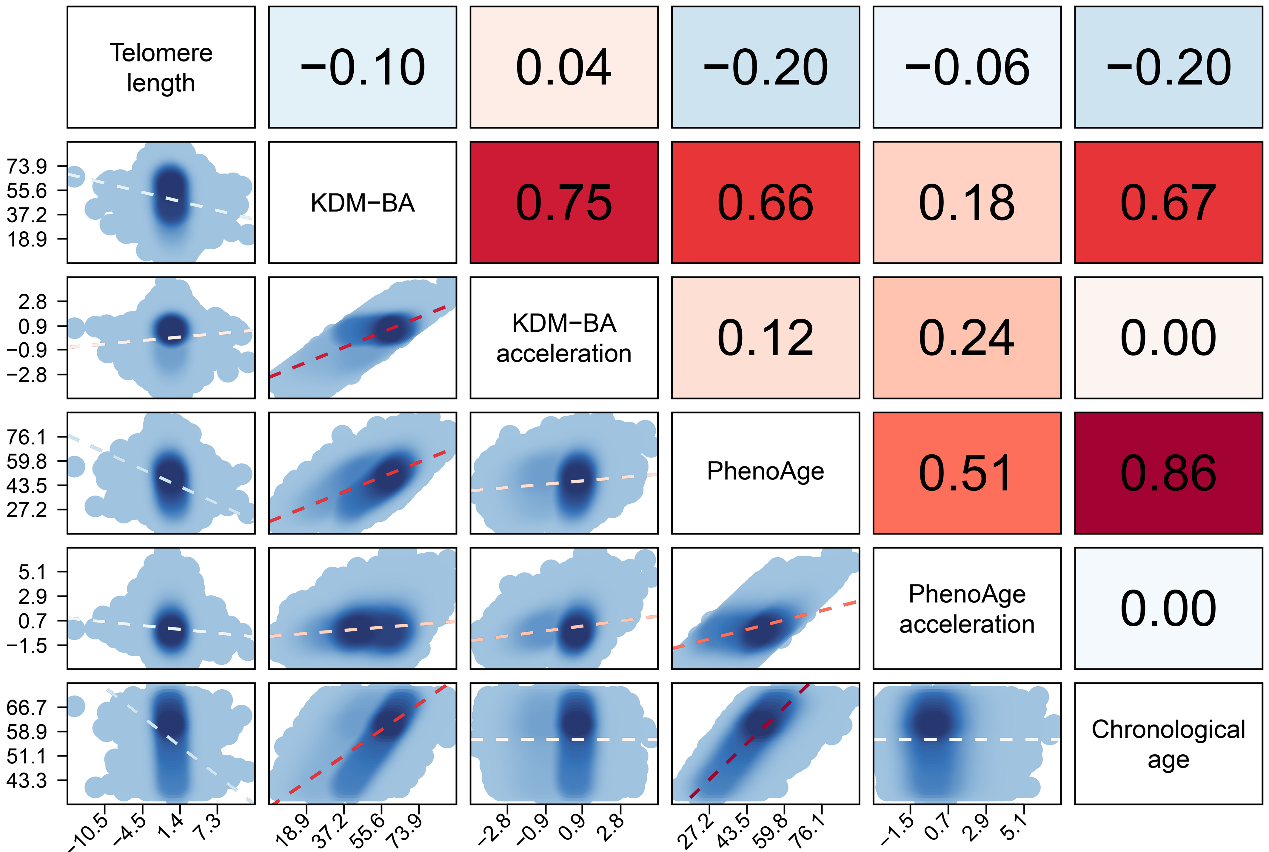


Abbreviation: KDM-BA, Klemera-Doubal method biological age; PhenoAge, phenotypic age;.

**Supplementary Fig. 3**. Sensitivity analysis for association between biological ageing and the risk of abdominal aortic aneurysm in the competing risk model


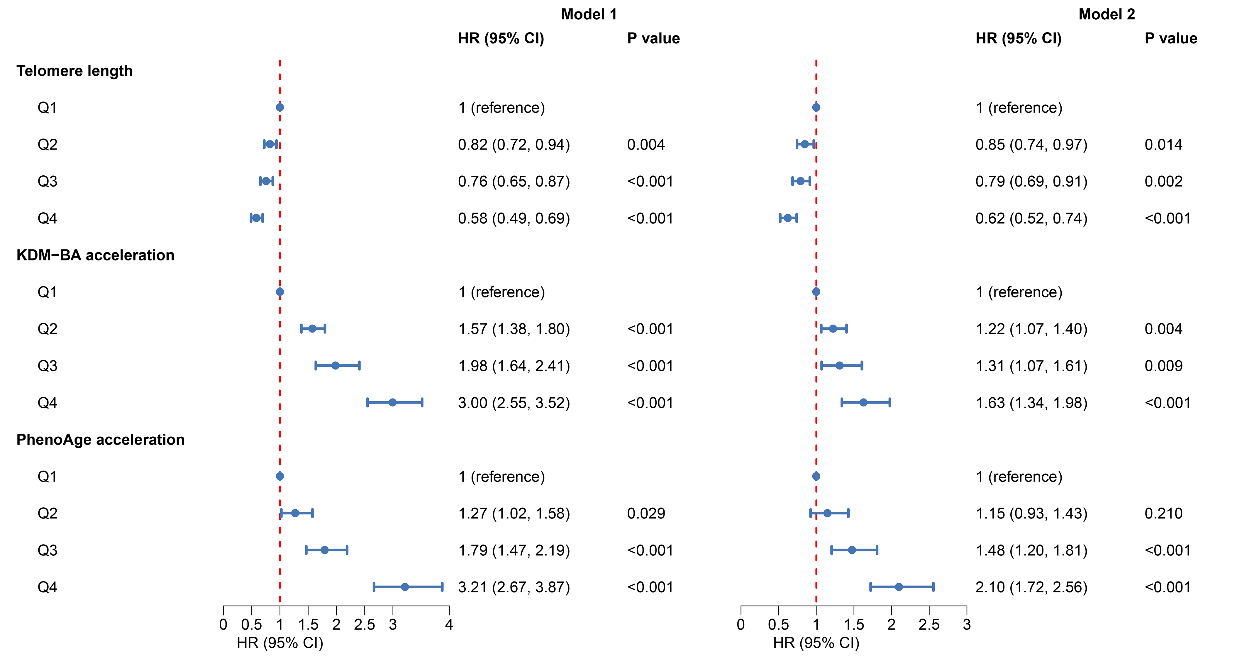


Model 1 adjusted for age, sex, ethnicity, Townsend deprivation index, education levels.

Model 2 adjusted for model 1 plus body mass index, C-reactive protein, smoking and drinking status, physical activity, sleep and dietary patterns, history of hyperlipemia, hypertension, diabetes mellitus.

Abbreviation: KDM-BA, Klemera-Doubal method biological age; PhenoAge, phenotypic age; HR, hazard ratio; CI, confidence interval.
